# Modeling intentionality in the human brain

**DOI:** 10.3389/fpsyt.2023.1163421

**Published:** 2023-08-09

**Authors:** Orestis Giotakos

**Affiliations:** Independent Researcher, Athens, Greece

**Keywords:** intentionality, intentional system, failure of intentionality, mental disorders, brain, modeling, philosophy, neuroscience

## Abstract

This paper is focusing on a rather neglected issue that concerns both aspects of philosophy and neurobiology in relation to the concept of intentionality. Intentionality is concerned with the ‘directedness’ or ‘aboutness’ of mental phenomena towards an object. Despite the fact that in philosophy both concepts of aboutness and directedness are conceptually identical with intentionality, a careful neuroscientific approach can demonstrate that these two phenomena represent two distinct conceptual and neurobiological aspects of intentionality with complementary functions. We described the interaction between a series of intentionality and pathogenetic psychobiological factors, the corresponding brain topography, and the resulting clinical manifestation and psychopathology. A permanent failure of intentionality dominates in psychosis, which includes an inappropriateness of the intentional object or connection, from the outset, or even from the prodromal phase of the disorder. Affective disorders may result from imprecise interoceptive prediction error signals, due to a confused identification of the intentional object. In suicidal patients there is an emotional intentionality failure, characterized by an absence of intentional object or a loss of conscious access to normal intentional objects. We may model an ‘intentional system’ as a higher order system, with a monitoring and regulatory role attributed to the brain and behavior. Also, we may consider mental disorders as the result of a radical disruption of intentionality, due to an inappropriateness or lack of the intentional object or due to an inappropriate connection in some points of the suggested brain pathways of intentionality.

## Introduction

The term ‘intentionality’ was coined in the 13th century by St Thomas Aquinas, with the aim of Christianizing Aristotle’s biological concept by utilizing this concept to describe the process by which human beings and animals thrust their existence into the world. According to Brentano, intentionality is considered to be the directedness of mental phenomena towards an object (1). Husserl argues that every aspect of consciousness contains intentionality, in the sense that all these aspects are always directed towards something and are always about something (2), whilst according to Heidegger, the subject is structured with intentionality within its own self (3). Spinoza emphasizes that every being by its very nature tends to maintain its existence, by naming this tendency ‘conatus’, a Latin term that denotes will and appetite. In his philosophy, “what a thing is” becomes identical with its power, its energy, its force of life (4). According to Searle, intentionality is that characteristic of the mind with which mental states are directed *at* or deal *with* (about) or refer *to* or are aimed *at* states of the world (5). His theory also raises the problem of the intentionality of perception, by using the expression relating to the “experience of” and emphasizing that the preposition “of” in the “experience of” is precisely the “of” in the “intentionality of.”

Human intentionality is closely associated with consciousness and agency. Intentionality appears to have strong connections to both consciousness as well as evolutionarily selected functions, by constituting the initiation, construction, and direction of behavior within the world. Consciousness consists in the proposition that X is a state of consciousness if and only if there is “something it is like” for the organism to be in that state (6). Several theories of consciousness are based on a connectionist approach, having a model of large webs with interacting neurons, fundamental to understand brain functionality, cognition, and behavior, while recently have been identified fundamental principles common to theories of consciousness, both classical and quantum (7). The *Husserlian* correlation between acts of thought – noesis – and intentional objects of thought – noema – becomes the first step in the constitution of analyses of consciousness. The feeling that one is the owner of his or her mental states consists in a contingent relation between consciousness and its intentional objects (8). We will say that intentional states (intentionally) represent their intentional contents (9). In saying that the mind is intentional, phenomenologists imply that the mind is relational. ‘Being in the-world’ (Heidegger) and the ‘lived body-environment’ (Merleau-Ponty) are different ways of articulating this kind of relation. The later emphasized the body as the primary site of knowing the world, demonstrating a corporeity of consciousness, as much as an intentionality of the body (10).

## The naturalistic theory of intentionality

Naturalizing intentionality in terms of tracking or functional roles is one of the most important goals in philosophy of mind (9). ‘Naturalization’ and ‘naturalism’ are frequently conflated with ‘reduction’ and ‘reductive physicalism’. Many philosophers think that metaphysical naturalism entails meta-philosophical naturalism, i.e., the view that philosophy is continuous with the empirical science (11). According to the metaphysical naturalism, the scientific version of naturalism, the view that human mind is part of the natural world, necessarily entails epistemological naturalism, i.e., the view that natural scientific understanding is the only way of making things in the natural world intelligible (12).

Neander’s main assumptions is that most intentionality is ultimately derived from the underived (or original) intentionality of nonconceptual sensory-perceptual representations and perhaps some core concepts (13). Millikan’s theory (14) explains intentionality in mainly biological and teleological terms within this context, by using the interpretive sources of natural selection. What thoughts and desires are ‘about’ is ultimately determined by why they have been chosen, namely what advantages they provided to ancestors once upon a time. Central to these teleological approaches is the idea that the production of mental representations needs to benefit the organism if not always, at least in some occasions. This is the rationale behind the very existence of the representational mechanisms historically carried over generations of organisms. However, in a teleological and behavioral view, free will is not something people essentially have or do not have, while the kind of actions that are typically seen as free are the same as those seen as self-controlled (15). The various degrees of freedom depend on our knowledge of the causal goings-on that affect us. The more we know, such as the various physical, biological, psychological, or sociological factors that affect our lives, can be considered as an expression of our rationality and consequently of our freedom (12, 16).

**Figure 1:**
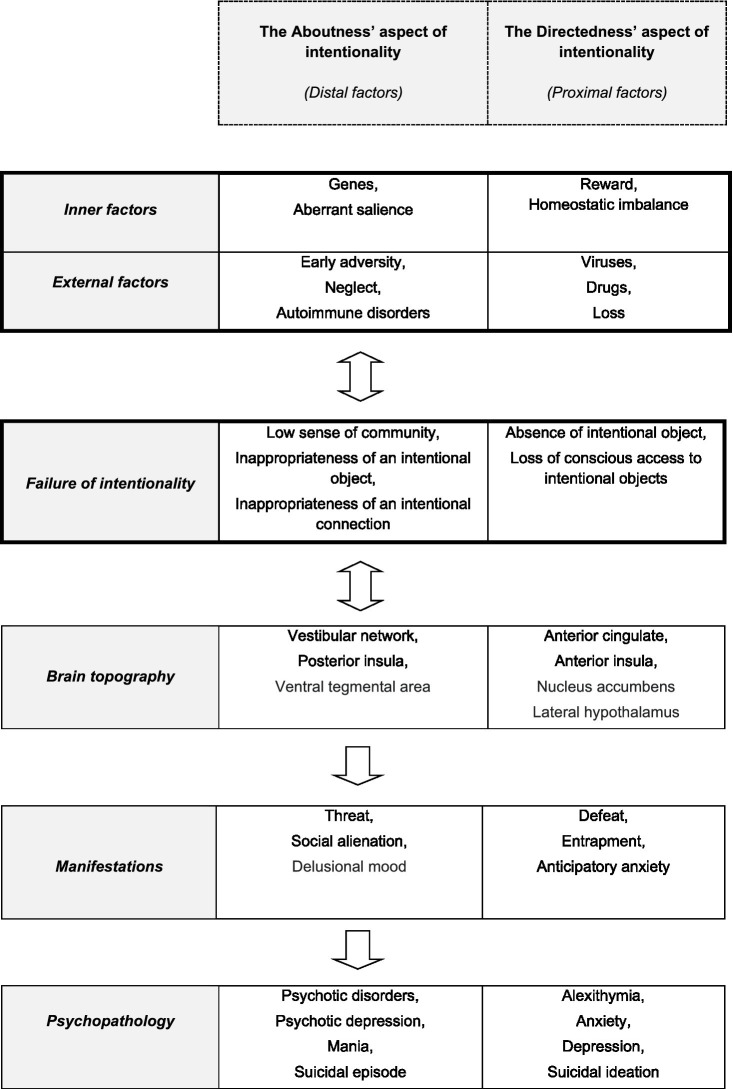
The interactions between the environmental and the pathogenetic psychobiological and intentionality factors, following by the involved brain regions, as well as the resulting clinical manifestation and psychopathology.

Biologists view every form of intentionality as the result of evolution through natural selection. ‘Intrinsic intentionality’ and ‘nano-intentionality’ have been proposed as microscopic forms of aboutness, which are innate in eukaryotic cells that include a goal-directed ability to adaptively respond to new situations. This nano-intentionality is a necessary precondition for the capacity of human organisms to have full-blooded intentional thoughts (17). A recently suggested *estimator theory* (18) is based upon an internal probabilistic process that is contained in every organism, which can estimate the evolutionary robustness of each organism. According to this theory, a naturalistic theory of intentionality must describe and explain the following properties of intentionality: (a) Directedness must be “many-to-one,” i.e., a single entity may simultaneously be the target of many prepositional pieces; (b) Directedness may be “one-to-many,” i.e., a simple prepositional piece may simultaneously be the target of multiple entities; and (c) a capacity should exist for contingent errors, as well as systematic errors, which implies that a prepositional object must have the capacity to misrepresent (see Figure 1).

## Intentionality and domain specificity within the brain

Human intentionality has a profoundly biological origin, whereby people have to produce intentional states (19–23). At micro-level, the so called ‘micro-intentionality’ or ‘nano-intentionality’ of cells, as a form of ‘intrinsic intentionality’, is extended by analogy in hearts or kidneys, which can have “derived” intentionality (17). In accordance with the theory of ‘autopoiesis’ (24), the essence of ‘perspectivity’ is a relational process taking place between the system and its world, characterized by relationality and transcendence (25). At macro-level, we are thinking about ‘macro-intentionality’ and the emergence of collective knowledge and cumulative culture in animals, humans and machines (26). Active exploration of an organism’s spatial environment and predation were the most important selective pressure to create learning (27), while the ‘first’ brains were formed and became powerful prediction machines (28). Findings in human brains indicate that the Default Mode Network, perhaps centralized to the dmPFC, primes the intentional stance to social stimuli. Default Mode Network activity in between moments of cognitive activity seems to be the biological basis for the powerful human tendency to adopt the intentional stance (29).

Despite the fact that in philosophy the concepts of ‘aboutness’ and ‘directedness’ are conceptually identical with ‘intentionality’, a careful neuroscientific observation is in a position to indicate that these two phenomena represent two distinct components with a complementary function. It has no relation with the proposed views of intentionality, such as linguistic, derived or original intentionality (9), but with inner parts or structure of intentionality. In a recent paper, using relative neuroscientific findings (19, 30–33), we provided an overview of possible pathways of an ‘intentional system’ in the brain, according to the conceptual and neurobiological traces of ‘aboutness’ and ‘directedness’ in the brain and in the behavior (34) (Table 1).

**Table 1:** The main conceptual features of the aboutness’ and directedness’ aspects of intentionality.

	The aboutness aspect	The directedness aspect
Design	About something	Toward something
Function	Being in the world	Towards the world
Phenomenology	Presence	Motility
Physiology	Homoeostasis	Reward
Micro-intentionality	Autopoiesis	Tropism
Macro-intentionality	We-intentionality	Joint-intentionality
Ego/allo-centrism	Ego-centric	Allo-centric
Information Flow	Bottom-up	Top-down
Representations	Production of representations	Use of representations
Conscious level	Mostly non-conscious	Mostly conscious
The Self	Me	I
Causality	Predisposing – Distal Factors	Accelerating – Proximal factors

Firstly, we suggested a common conceptual and neurophysiological ground for both ‘intentionality’ and ‘conatus’, through the study of the complementary and interacting functions of aboutness and directedness. We hypothesized an ‘aboutness sub-system’, which has been designed having the ability to ‘be about things’, to ‘predict’ and ‘be present’ in the world. Also, it has been designed having the ability to support ‘homoeostasis’, through an ‘ego-centric’ mode of operation. On the other hand, the ‘directedness sub-system’ has been designed so as to be able to direct existence “toward” the world, by having a close relationship with the conative elements of appetite, creation and renewal. Also, to support ‘reward’ behavior, in parallel with an ‘allo-centric’ way of function.

The vestibular system is the first sensory system to begin development *in utero*, and it is the first fully developed by the eighth month of intrauterine life (35). It is a sensory system that never “sleeps,” since gravity impacts any movements in relation to the world around us. Anecdotal observations indicate that whirling by Sufi semazen-artists or mindful movements by yogic practitioners profoundly impact vestibular stimulation, supporting a state of presence and tranquility (36). Conceptually, ‘aboutness’ seems to be strongly associated with the vestibular network. We hypothesize that aboutness sub-system is primarily based upon the vestibular network and the interoceptive system, where thalamus and insula are the main hubs. The directedness sub-system is mainly based upon the dopaminergic network and the orexigenic/anorexigenic neurons connected to hypothalamus (34).

An intentional transfer of information is central to human communication (22). The parietofrontal pathway relays bottom-up target information for sensory-driven reflexive movement, while the frontoparietal pathway relays top-down target information that is cognitive rule-based (37). Also, the predictive processing framework indicates existence of two types of prediction error neurons: positive prediction error neurons, subtracting a top-down prediction from the sensory input, and negative prediction error neurons, subtracting sensory input from the top-down prediction (38). In a similar way, and having in mind the rest aspects of intentionality function, we hypothesize the aboutness sub-system has a bottom-up direction of information flow, running mostly in a non-conscious way. On the other hand, the information flow is top-down in the directedness sub-system, running mostly in a conscious way (34). It has been proposed that some of consciousness in unreflective actions can be characterized by intentionality being inhibited. In these cases of ‘inhibited intentionality’ an agent feels a diminished sense of authorship in relation to self-understanding (39).

Dennett emphasized that the intentional stance of things is a strong position, which could be developed into a reliable *intentionality theory* (40). Based on the philosophical, social and neuroscientific findings, as well as the hypothesized aspects, features and functions of intentionality, we may suggest an *intentional system*, as a higher order system, which plays a supervisory role in both the brain and behavior. The *cybernetic model* (41) emphasizes on *intention* and includes the lack of awareness of ones’ goals, which leads to disorders of willed action, the lack of awareness of intention, which leads to movement disorders, and the lack of awareness of intentions of others, which leads to paranoid delusions and hallucinations. The theories of metarepresentation involves mainly output mechanisms, like the frontal cortex, while the input mechanism involves posterior brain systems, including the parietal lobe (42). The inferior part of the posterior parietal cortex seems to be a crucial area for the updating of information in the working memory and the BA *46 & 9/46* encode it into an “abstract/symbolic form,” in order to achieve the controlled monitoring in the active mnemonic process. This system has the capacity to hold symbolically coded information in an active state, in order to supervise the between them relation and their relation with the intended programmed behavior (43).

At the social level, Mead develops William James’ distinction between the ‘I’ and the ‘Me’. He postulated ‘Me’ is the social self and the ‘I’ is the response to the ‘Me’. The ‘I’ is the response of an individual to the attitudes of others, while the ‘Me’ is the organized set of attitudes of others which an individual assumes. ‘Me’ is the object and ‘I’ is the subject. ‘Me’ is the known and ‘I’ is the knower (44). Extending, we can hypothesize that ‘aboutness’ is corresponding to ‘Me’, which is an organized set of attitudes, and continuously enriching by the ‘directedness of ‘I’, i.e., the response of an individual to the attitudes of others. Moreover, we can also hypothesize that ‘aboutness’ contains the priors, including the predisposing, i.e., the distal factors, while ‘directedness’ contains the accelerating or proximal factors of the self.

## Modeling the failure of intentionality in the human brain: implications for mental disorders

### Inappropriatness of an intentional object or connection

The mind functions normally to the extent where its intentional objects and connections are appropriate. Bolton speculated whether mental disorders could be considered as a group of radical failures of intentionality. He pointed that a failure in intentionality that is due to the inappropriateness of intentional object or a corresponding connection, or even the absence of an intentional object, can lead to a mental disorder (45, 46). In the philosophy of mind, ‘aboutness’ is considered to be synonymous with intentionality, referring to the concept where a phrase, image or action is *on* or *about* a certain object. If human intentionality is closely related to consciousness and agency, what could play the role of a specific network in the brain for the aboutness formation? Moreover, could an inappropriate intentional object or connection of that system result in a mental disorder? In the Table 2 are *summarizing* the failure of intentionality, the corresponding brain regions, the resulting clinical syndromes, and the possible therapeutic implications.

**Table 2:** Summarizing the failure of intentionality, the corresponding brain regions, the resulting clinical syndromes, and the possible therapeutic implications.

	The aboutness aspect	The directedness aspect
Failure of Intentionality	Low sense of community, Inappropriate of an intentional object, Inappropriate of an intentional connection	Absence of intentional object, Loss of conscious access to intentional objects
Brain topography	Vestibular network, Insula, Ventral Tegmental Area, Default Mode Network	Nucleus Accumbens, Anterior Cingulate, Insula, Amygdala, Lateral Hypothalamus
Clinical manifestations	Threat, Social alienation, Delusional mood	Defeat, Entrapment, Anticipatory anxiety
Psychopathology	Psychotic Disorders, Bipolar Disorders, Psychotic Depression, Suicidal episodes	Alexithymia, Panic Disorder, Anxiety, Depression, Addiction, Suicidal ideation
Therapeutic implications	Social cohesion, Early intervention, Diet, Psychoeducation, Resilience support	Meta-emotion strategies, Mindfulness awareness meditation, Body psychotherapy, Drug policy

For Jaspers, schizophrenia principally refers to the loss of the basic sense of ownership and agency of one’s own experiences, thoughts, or actions (47). Corollary discharge, is a basic neurophysiological mechanism that is involved in sensory prediction and contributes to the distinction between self-generated and externally generated sensory information (48). This helps the organism to establish the difference between self and non-self (49) and to prevent self-induced desensitization (50). It has been argued that delusions and hallucinations is the result of the failure of corollary discharges to suppress self-generated inputs, resulting in the absence of “feelings of agency” in the *ego-centric* system, and in the compensatory enhancement of *allo-centric* priors (51). In addition, the *salience network* plays an important role in stimulus processing, attention, and the switching from the *default network* to the *executive system*. The structures involved are the *anterior insula* and the *anterior cingulate cortex*, whilst dysfunction of this network is considered to be involved in the development of various mental disorders (52, 53).

The *intentional content* of the visual experience in the *Muller-Lyer illusion lines* example comes into conflict with and is overridden by the intentional content of our beliefs. The perceiver has the same experience in the case of hallucinations, but there is no intentional object present (5). Schizophrenia patients showed a severe loss of the ability to detect internal bodily signals and to attribute them to themselves, while interoceptive accuracy was associated to patients’ positive symptomatology (54). The distinction between the correct and incorrect representation is often regarded as a central normative distinction and a capacity to *misrepresent* is often considered to be essential for representation: if there is no possibility of misrepresentation, there is no representation. This is connected to concerns with non-existent objects, where a capacity to misrepresent amounts to a basic capacity to represent non-existent objects (55). We may argue that a loss of both may exist in psychosis: namely, the intentional object and the capacity to misrepresent (18, 34). We may moreover suggest that a permanent disturbance of intentionality dominates in the negative syndrome of schizophrenia, which may include the inappropriatness of an intentional object or connection, from the outset, or even from the prodromal phase of the disorder (34, 56, 57).

The subjective experience in psychosis seems to be rooted in the disturbance of intentionality and diminished sense of agency (58), while their referential and persecutory ideation motivates inappropriate mentalizing when objective cues of intentionality are absent (59). Psychotis exhibit a striking bias on over attribute intentionality, and especially an inability to inhibit the automatic attribution of intentionality (60). Schizotypy was found to be associated with perceiving ambiguous actions as intentional, particularly in social contexts (61), Also, a hyper-intentionality state has been found to be prominent in psychosis and a hypo-intentionality state in autistic spectrum disorders (62). Psychotics show an amplified bias for intentionality, or they have trouble controlling this bias? (30). Or, do they lose conscious access to the normal intentional object of their emotional experience, providing a delusional explanation for the absent intentional object? (45, 46). Theories of self-monitoring and error-checking agree with the theory concerning the use of a salience network. In psychosis, the initial hyperactivation of the salience network is likely followed by the hyper-activation of the default mode network and subsequently by the suppression of the salience and attention network (63).

The intentionality bias in psychosis, particularly in social contexts, can be better understand through the theories on ‘shared intentionality’. The Japanese philosopher Watsuji developed the concept of ‘betweeness’, reporting that the character and structure of intentionality is constitutively dependent upon an individual’s deep integration with their sociocultural environment (64). According to Searle, there could not be any social reality without collective intentionality and there could not be any *collective intentionality* without a pre-intentional *sense of community*. Shared intentionality, in a synonymous sense to collective intentionality, is described as the power of the mind to share mental states, such as feelings, intentions and beliefs with others (65). Shared intentionality, sometimes called “we” intentionality, refers to collaborative interactions in which participants have a shared commitment for pursuing a shared goal (65). Shared intentionality arises within human ontogeny and provides the basis for other capacities that are also apparently unique to humans (66). With the maturation of particular brain regions in infants we can see the emergence of the mental state of the idea of me – the “I know I know” and “I know you know,” which then subsequently allows for “I know you know I know” (67). Two types of implied intentionality have been proposed: (a) ‘Joint intentionality’ relies on the agents’ mentalizing abilities, such as mind reading and the ability to factor in their partner’s intentions, during an action planning. (b) ‘We-intentionality’ relates to the ability to perceive others as members of a group and to adopt the group’s expectations, a process found to be impaired in psychosis, while in severe autism spectrum disorder, both forms of shared intentionality found to be impaired (68).

However, in real world changes in many outcomes are rarely linear, and failures of intentionality in psychosis seem to occur through a non-linear and multifactor fluctuating pattern. Brain systems reflect components of an *intentional system*, where, as non-linear dynamics, can explain the complex behavior of such fuzzy systems (69). Recently we suggested that in psychosis there exist a failure of intentionality, due to a constant inappropriateness of an intentional object or connection (56). Using the *cusp catastrophe model* paradigm (70), we explained why relatively small changes in a parameter can result in catastrophic changes in the system state, while the sudden collapse can be explained by an already fragile system, rather than by a novel stressor. For example, the dysmyelination-induced delays in psychosis may cause a discrepancy in sensory feedback mechanisms, resulting in chronic prediction error, and in a generally fragile system. Treatments that preserve white matter integrity or ameliorate white matter disruption may enhance information-processing and functional outcome in psychosis (71).

### The loss of intentional object

Intentionality is also considered to include the ‘directedness’ of mental phenomena towards an object. Could it possible that the loss of the intentional object can result in a mental disorder? Also, could a specific place exist for ‘directedness’ in the brain?

The *reward circuits* could represent such functions. These include the amygdale, which is associated with emotional learning, the ventral tegmental area, which contains dopaminergic neurons and signals motivation and reward seeking behavior, the nucleus accumbens, which is centrally involved in reward learning, and the lateral hypothalamus, which completes these reward signals, by uniting the homeostatic system with the hedonic system (72). Reward circuit starts from the *ventral tegmental area* and projects into many parts of the limbic system, such as the amygdala, the orbitofrontal cortex, the anterior cingulate and the nucleus accumbens (73, 74). The reward-dependent plasticity of basal ganglia neurons is caused by inputs from dopamine neurons located in the substantia nigra pars compacta and ventral tegmental area, which encode the ‘reward prediction error’ (75). As a key node in the reward pathway, the nucleus accumbens is important for determining motivation-to-action outcomes (76). The pleasure response is related to the mesolimbic dopaminergic system, which is involved in eating, in the use of psychoactive substances and in sexuality. The dopamine-guided plasticity would guide animals to choose actions that lead to better rewards (77). According to Pankseep, the brain contains a system of exploration and search (*seeking system*) that is responsible for the ability to have an urge towards something and this system sets the ability to open a primordial time horizon (78). We may consider that appetite and feeding, combining both aboutness and directedness sub-systems, are regulated by two interacting systems: the *homeostatic system*, which ensures that the individual receives sufficient calories for survival, and the *hedonic system*, which regulates the pleasure and reward aspects of feeding (79).

Another field of interest may be the networks associated with *predictive processing*, a function of vital significance for survival. Predictive coding appears as a universal evolutionary pathway and is constantly modified by environmental and internal mental information. The *insula* plays a role in not only error estimation but also in the updating of the probabilities of an outcome. It has been described as a specific hub of the autonomous, emotional and cognitive integration, and is associated with a range of stimuli, such as cognitive, emotional, olfactory, interoceptive and pain sensations (21, 33, 80). The *interoceptive predictive coding* supports a new view regarding emotions and the significance of the *interoceptive system*. It has been hypothesized that chronic anxiety is due to an increased interoceptive predictability of error signals, whilst the disrupted interoceptive predictive coding may be causally related to many psychiatric disorders (81). Interoceptive sensitivity is correlated with emotional stability. Atypically high interoception has been argued to characterize both panic disorder and anxiety syndromes, while poor interoceptive sensitivity has been observed in individuals with depression and schizophrenia (54).

It would appear that moods are pre-intentional states, which constitute the background within which the prepositional directed emotions target the objects. It is considered that the emotions shape the expectant structure of experience, which is a structure that makes intentional, mental, and bodily actions possible. This is why these phenomena have been suggested as background feelings, or possibility structures, or styles of anticipation of experience (82). *Bodily feelings* have intentional objects within the body, whereas *feelings towards* have an intentional object outside the body (83). In the case of depression, grief-pang is associated with the feeling of grief, which is apparently manifested through a process of “borrowing” the lost intentional object of grief (84). Furthermore, alexithymia, characterised by both atypical interoceptive sensitivity and loss of intentional object, has been found to co-occur with a number of affective disorders (85). Alexithymia and dissociation have been consistently linked in the literature, particularly in psychiatric populations. Both arise from a disconnection between conscious aspects of self-experiences and perceptions at both the mental self and bodily levels (86). Moreover they have been linked with a broader loss of emotional color, while psychopathology may result from imprecise interoceptive prediction error signals, due to a confused identification of the intentional object (21, 80).

In the case of suicidality, ‘entrapment’ was found to be the strongest predictive indicator for suicide (20, 57, 87). We may suggest that in suicidal patients there is an *emotional intentionality failure*, characterized by an absence of intentional object or a loss of conscious access to normal intentional objects. The suicidal action is accordingly the result of despair, loss of emotional intentionality, and entrapment, in a psychological environment with vague feelings (20). In our relative paper (88) we proposed an intentionality failure theory for suicidality, pointing that theories of emotional intentionality can enrich understanding of the transition phase from suicidal ideation to suicidal action. Taking the idea from the cusp catastrophe model (70), we demonstrated a multifactor fluctuating pattern of suicidality where intentionality failure was strongly tied with entrapment. If distal risk of suicidality is low, then proximal risk will be linearly related to a suicidal episode, but, if distal risk is high, then proximal risk is nonlinearly related to suicidality, and small changes in proximal risk predict a sudden lapse. It should be noted that people who commit suicide without having a psychiatric diagnosis can also be fitted in the proposed model, since the majority of people who die by suicide have never seen a mental health professional (89). This model suggests that strategies for sustainable management of such cases should focus on maintaining resilience (88). Also, different psychotherapeutic treatments focusing on the body (90), such as, yoga and mindfulness (91), dance therapy (92), dance moving therapy (93), or mirror exposure/gazing (94), can help patients, to rediscover and define their lost intentional object.

### Epilogue

This manuscript is focusing on a rather neglected issue that concerns both aspects of philosophy and neurobiology, relating to the concept of intentionality. Although it is difficult to identify the associations between the observational feature of phenomenal concepts and the theoretical feature of intentional concepts (32), we may suggest an *intentional system*, as a higher order system, which plays a supervisory role in both the brain and behavior. A series of potential associations of this system in the brain have been presented in this manuscript, by observing the conceptual and neurobiological traces of aboutness and directedness. Additionally, a series of mental disorders have been suggested as the result of the failures of this neurophenomenological construction. We may consider that clarifications of the relationship between these associations shall further assist with comprehending the interaction between the brain and the mind, as well as our extension and interaction in the world.

The present paper is constrained by the well-known limits regarding suggested theoretical models, suffering from issues related to consistency and generalizability. Many parts of the paper are only theoretical and they need further support by clinical and preclinical studies, since these are philosophical ideas which have not or cannot be adequately supported by neuroscientific evidence.

However, when more scientific evidence will be gathering, we could be in a position to study ‘disordered intentionality’ in a dimensional framework, as a transdiagnostic biobehavioral system, according to the Research Domain Criteria (RDoC) or/and the Hierarchical Taxonomy of Psychopathology (HiTOP), which have the potential to inform the development of a unified, dimensional, and biobehaviorally-grounded psychiatric nosology (95). Finally, having in mind the etiological and diagnostic heterogeneity of psychiatric disorders, as well as the emphasis of the recent treatment approaches on the potential of brain-circuit-based interventions for precision psychiatry (96), we may think that the perspective of targeting brain circuits related to intentionality could offer hopeful treatment approaches in the future.

## Data availability statement

The original contributions presented in the study are included in the article/supplementary material, further inquiries can be directed to the corresponding author.

## Author contributions

The author confirms being the sole contributor of this work and has approved it for publication.

## Conflict of interest

The author declares that the research was conducted in the absence of any commercial or financial relationships that could be construed as a potential conflict of interest.

## Publisher’s note

All claims expressed in this article are solely those of the authors and do not necessarily represent those of their affiliated organizations, or those of the publisher, the editors and the reviewers. Any product that may be evaluated in this article, or claim that may be made by its manufacturer, is not guaranteed or endorsed by the publisher.
